# Rare cases of medulloblastoma with hypermutation

**DOI:** 10.1002/cnr2.1521

**Published:** 2021-08-05

**Authors:** Aditi Bagchi, Ian Beddows, Albert Cornelius, Giles W. Robinson, Scott D. Jewell

**Affiliations:** ^1^ Van Andel Institute Graduate School St. Jude Children's Research Hospital, Spectrum Health Helen DeVos Children's Hospital Grand Rapids Michigan USA; ^2^ Division of Pediatric Hematology and Oncology Spectrum Health Helen DeVos Children's Hospital Grand Rapids Michigan USA; ^3^ Division of Neuro‐oncology, Department of Oncology St. Jude Children's Hospital Research Hospital Memphis Tennessee USA; ^4^ Bioinformatics and Biostatistics Core Van Andel Research Institute Grand Rapids Michigan USA; ^5^ Pathology and Biorepository Core Van Andel Research Institute Grand Rapids Michigan USA

**Keywords:** hypermutation, medulloblastoma, MMRD, POLE‐Mutations, SHH‐Medulloblastoma, signature 10

## Abstract

**Background:**

Medulloblastoma is the most common malignant brain tumor of childhood and is considered a tumor with low mutational burden (~1 Mut/Mb). Therefore, though the medulloblastoma genomes have been extensively characterized in literature, reports on potential hypermutations and underlying mutagenic processes in medulloblastomas are limited.

**Aim:**

In this report, we studied the landscape of mutational burden in primary and recurrent medulloblastoma. Furthermore, we wanted to understand the differences in underlying mutagenic mechanisms in medulloblastoma with low and high mutational burdens.

**Methods:**

Fifty‐three primary and recurrent medulloblastoma genomic sequence were downloaded from the European Genome Archive as BAM files. Thirty‐three cases were obtained from formalin‐fixed paraffin‐embedded tissues from pathology diagnostic archives of Spectrum Health and Cooperative Human Tissue Network. Somatic mutations were called using Mutect2, following best practices guidelines for Genome Analysis Toolkit V4. Mutational signatures were analyzed using *deconstructSigs*.

**Results:**

We identified nine medulloblastoma cases with high mutational burden (>5 Mut/Mb). Of them, five cases met the criteria of hypermutation (>10Mut/Mb), two of the five tumors had canonical mutations in the *POLE* proof‐reading domain, where a large proportion of mutations in these tumor genomes contributed to signature 10. The hypermutated cases also demonstrated mutational signatures 14, 15, and 21, indicating the role of mis match repair deficiency in their mutagenesis. Of the four known molecular subgroups in medulloblastoma–SHH, WNT, Group 3, and Group 4—both the *POLE*‐mutated cases belonged to the SHH subgroup. This report identifies rare cases of hypermutation in medulloblastoma driven by defects in DNA repair mechanisms.

**Conclusion:**

Hypermutation in medulloblastoma can impact therapeutic decisions, especially at recurrence in otherwise fatal high risk SHH‐medulloblastomas. A defect in DNA repair leading to SHH ‐medulloblastoma is yet another important mechanism that should be further investigated in the genesis of these tumors. Therefore, this report provides important scientific and clinical rationale for future research looking for incidence of hypermutation in large cohorts of medulloblastoma patients.

**Abbreviations**
GATK: Genome Analysis Tool Kit
EGA: European Genome Archive
Mut/Mb: mutations per megabase
MB: medulloblastoma
SMI: small molecule inhibitor
TMB: tumor mutational burden
WGS: whole genome sequencing
MMRD: mismatch repair deficiencies

## 1 INTRODUCTION

Tumor mutational burden (TMB), defined by the number of nonsynonymous DNA mutations per megabase (Mut/Mb) of the genome's coding region, is a potential biomarker of tumor response to immune checkpoint inhibition.^1^, ^2^ High TMB tumors generate neoantigens triggering an antitumor cytotoxic T‐cell response attenuated by immune checkpoints, which have been studied in various tumors including brain tumors (e.g., high‐grade gliomas^3^, ^4^). Medulloblastoma (MB) has been extensively analyzed in genomic, transcriptomic, and methylation studies classifying MB into molecular subgroups—Wingless (WNT), sonic hedgehog (SHH), Group 3, and Group 4—by clinically relevant and unique transcriptional, genomic, and epigenetic features.^5^, ^6^ In general, genomic studies have shown that childhood MB has a low TMB,^7^, ^8^ and, as such, these patients with MB are not considered as good candidates for immune checkpoint inhibition therapy. However, in this study we identify five tumors with >10 Mut/Mb and two of them with *POLE* mutations belonging to the SHH subgroup, suggesting high TMB does occur, albeit rarely, in medulloblastoma.

## 2 METHODS

Use of participants' tissues in genetic studies was approved by Institutional Review Boards of Van Andel Research Institute and Spectrum Health Helen DeVos Children's Hospital. Permission to download whole genome sequencing (WGS) data of 53 primary and recurrent MBs was obtained from the European Genome Archive (EGA). Thirty‐three cases were obtained from formalin‐fixed paraffin‐embedded tissues from pathology diagnostic archives of Spectrum Health and Cooperative Human Tissue Network. DNA was extracted from 50 to 100 μ of FFPE curls using the spin column‐based nucleic acid extraction protocol as published and manufactured by Qiagen for nucleic acid extraction from FFPE tissue (Cat No. #56404). SureSelect^XT^ Clinical Research Exome V2 (Agilent Technologies) was used to capture exomes of tumor samples according to the manufacturer's protocol, with modifications based on degree of DNA fragmentation. Somatic mutations were called using Mutect2, following best practices guidelines for Genome Analysis Toolkit V4 (GATK).^9^ Copy number analysis was done using GATK v3.7 using their best practice workflow. See more details of methods in the result and Supplementary Sections. Supplementary Table 1 enumerates the computational tools used for data visualization and analysis in R computing environment.

## 3 RESULTS

**FIGURE 1:**
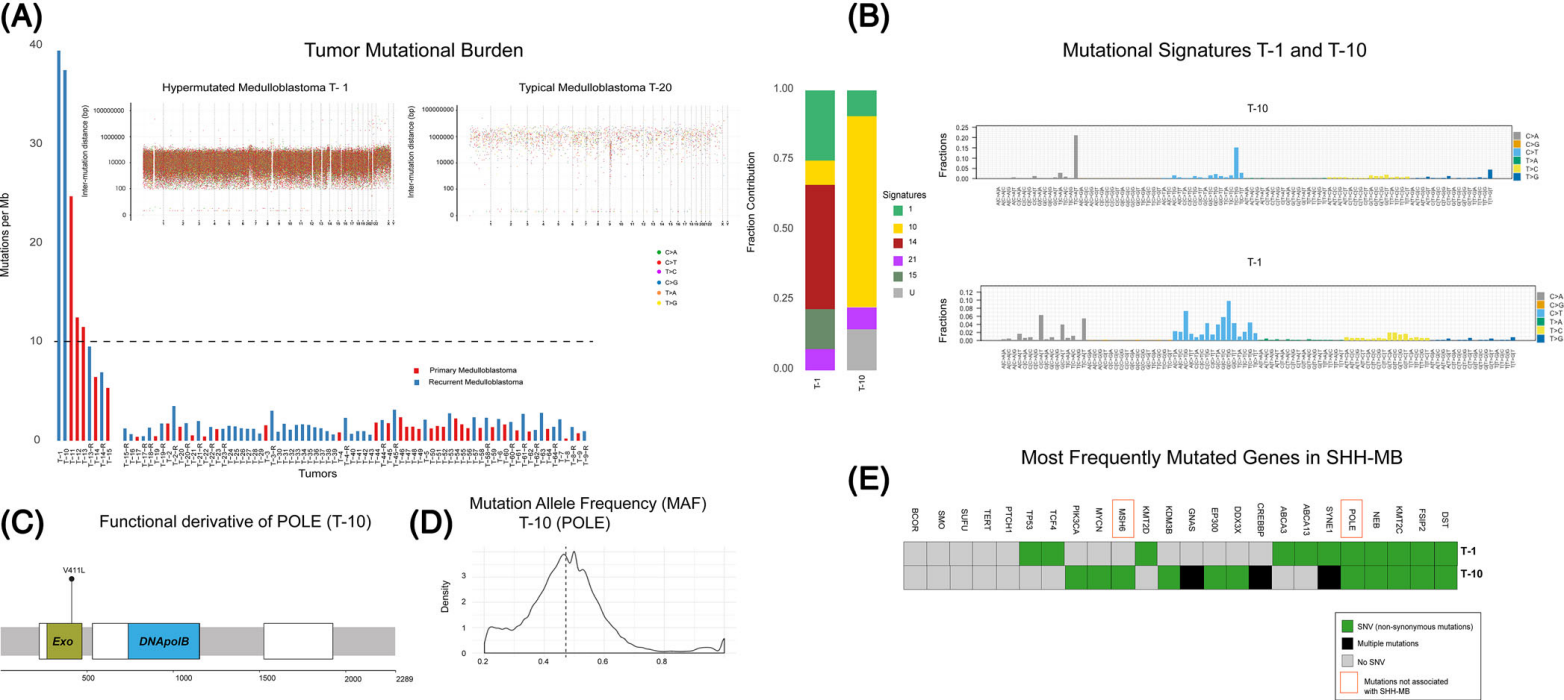
(A) Tumor mutational burden (TMB) of medulloblastoma (MB). The figure depicts TMB in the coding region. The Y‐axis depicts the total number of mutations per Mb, and the X‐axis depicts each individual tumor. Panels labeled “Hypermutated Medulloblastoma T‐1” and “Typical Medulloblastoma T‐20” are scatter plots of somatic mutations showing their locations on the X‐axis versus distance to other events on the Y‐axis. (B) Fractions of mutations contributing to different signatures in T‐1 and T‐10, including signature 10, which occurs in both tumors and fractions of 96 substitution types contributing to the signature profile of each tumor. The X‐axis depicts the 96 substitutions, and the Y‐axis shows the fraction of contribution. (C) Schematic plot demonstrating functional derivatives of the POLE protein and location of mutations identified in T‐10. (D) Distribution curve of mutation allele frequency (MAF), the dotted line depicts the MAF of the *POLE* mutation in the tumor. (E) Mutation (missense and nonsense mutations) detected in list of commonly mutated genes in SHH‐MB

TMB range in 86 primary and recurrent MB genomes and exomes was 0.2–39.5 Mut/Mb (mean 3.1 Mut/Mb; median 1.2 Mut/Mb; Figure 1(A)). Whereas most MB genomes (89.5%) had a low TMB, we identified 9 (10.5%) cases as outliers, of which 5 (5.8%) had TMB > 10 Mut/Mb, meeting criteria of hypermutated tumors^10^ (Figure S1A).

We evaluated mutational signatures to establish differences among low and high TMB cases.^8^, ^11^ Mutagenesis leaves marks on DNA (e.g., nucleotide substitutions), creating unique signatures. The initial definition of such mutagenic signatures reveals 21 signatures in human cancers.^12^ We cataloged 486 078 exonic and intronic mutations by nucleotide context (bases immediately preceding and following it, forming a trinucleotide). Using these cataloged trinucleotides (96 subtypes), we performed linear regression analysis using deconstructSigs^13^ to identify fractions of mutations contributing to previously established mutational signatures.^12^ Due to few mutations, exomes were not analyzed for mutational signatures.

We found signatures 1, 10, 14, 15, and 21 in hypermutated MBs (Figure 1(B)). However, no nonhypermutated MB had mutations contributing to signatures 10, 14, 15, and 21. Mutations in nonhypermutated MBs contributed to signatures 1, 3–6, 8, 9, 11, 12, 16, and 18–20, with a high prevalence of signatures 3 and 8 (Figure S1D).

In T‐10, with TMB of 37.5 Mut/Mb, 68% of mutations contributed to signature 10. This signature, characterized by C > A substitution in TpCpT and C > T substitutions in the TpCpG context, is specifically associated with loss‐of‐function mutations in the exonuclease or proofreading domain of *POLE*.^8^ T‐1, with a TMB of 39.5 Mut/Mb, had 9% mutations contributing to signature 10 (Figure 1(B)). We identified missense mutations p.R821C, p.D391E, and p.V411L in the *POLE* coding region in T‐1 and T‐10 (Table 1, Figure 1(C), and Figure S1B).

**TABLE 1:** Mutations in *POLE*

hg 38 position	CDS Change	AA change	COSMIC ID	Type of variant	Tumor ID
12:132673703	C ‐ > A	p. V411L	COSV57677068	Missense	T‐10
12:132676157	A ‐ > C	p. D319E	COSV57679046	Missense	T‐1
12:132665309	G ‐ > A	p. R821C	COSV57674926	Missense	T‐1

Abbreviations: CDS, coding sequences; AA, amino acid.

Presence of *POLE* mutations and signature 10 in a hypermutated tumor suggested that these *POLE* mutations were pathogenic. Furthermore, mutation in position 411 that cause amino acid Valine to leucine^10^ switch is known to be pathogenic. Therefore, we inferred that T‐10 with V411L mutation in the “proof reading” domain of POLE was hypermutated secondary to this mutation. Indirect evidence of pathogenicity of the POLE mutation was determined by calculating its mutation allele frequency (MAF). MAF was measured as total number of sequences reads observed matching a specific DNA variant divided by overall coverage at a given genomic locus and is a surrogate measure of the proportion of DNA in the tumor carrying the variant. The MAF of the *POLE* mutation was ~49% (Figure 1(D)), indicating that the mutation is a somatic heterozygous variant present in almost all tumor cells.

T‐1 had two different POLE mutations *D319E* and *R821C* (Figure S1B), both documented in the Catalog of Somatic Mutations in Cancer database. However, their pathogenicity and association with hypermutation are not well documented. Large percentage of somatic mutations contributed to signatures 14 and 15 in T‐1 (Figure 1(B)), these signatures has been functionally linked to mismatch repair deficiency (MMRD).^10^ We did not identify somatic or germline mutations in Mis Match repair (MMR) pathway genes mutations in T‐1. However, the most common cause of MMRD in human cancer, hypermethylation of MLH1 promoter,^14^ could not be tested due to unavailability of the biological specimen. Signature 21 was detected in both T‐1 and T‐10, signature 21, like signatures 14 and 15, is functionally associated with MMRD. Our mutational analysis revealed MSH6 mutation in tumor T‐10 (Figure 1(E)). Presence of signatures 10, 14, 15, and 21 in both tumors indicates two unique mechanisms of replication repair deficiencies driving hyper mutagenesis in these cases.

T‐1 and T‐10 tumors were obtained at the time of recurrence from 4.5‐ and 12‐year‐old males respectively. Both tumors belonged to the SHH‐MB molecular group, diagnosed using gene expression array,^15^ suggesting a possible predilection of hypermutator phenotype within SHH‐MB. In the absence of biological specimen, we attempted to identify other genomic features to validate the diagnosis of SHH‐MB. We generated a list of 22 genes that are frequently mutated in SHH‐MB from published literature.^16^, ^17^ Both tumors demonstrated mutations in several (Figure 1(E)) of these key genes. The list of 22 genes included several genes that encode histone acetyltransferase (HAT), we detected mutations in several HATs namely EP300 and CREBBP. The enrichment of mutations in HAT/HAT complex has been reported as a characteristic feature of SHH‐MB.^16^

No mutations in *CTNNB1* and other genes unique to WNT‐MB were identified in the hypermutated cases. Both T‐1 and T‐10 harbored molecular high‐risk features. Mutational analysis in T‐1 revealed TP53 mutation (Figure 1(E)), copy number analysis showed a 14q loss in T‐10 (Figure S2) which is known cytogenetic characteristic of SHH‐MB and is associated with poor prognosis.^18^

In the remaining hypermutated tumors, mutations in T‐13 and T‐13‐R contributed to signatures 1, 5, 12, and 16. Underlying mutagenesis driving signatures 12 and 16 remains unknown.

## 4 DISCUSSION

A recently published genomic analysis of 134 pediatric MBs for TMB found that most tumors had low mutational burden with 8/134 MBs (6%) displaying a mutational burden of 6–20 Mut/Mb.^19^ Our study, similarly, reports 9/86 MBs (10%) with a mutational burden of >5 Mut/Mb suggesting that although rare these cases do exist. Hypermutation associated with mutations in catalytic domain of *POLE* is even rarer in MB. To our knowledge, two other cases are reported: a 5‐year‐old child with non‐WNT, non‐SHH MB, with a germline *POLE* mutation and an adult patient with SHH‐MB with *POLE* mutation (V411L [same mutation detected in our reported case]).^16^, ^24^ In our cohort hypermutated tumors T‐1 and T‐10 with *POLE* mutations were obtained at the time of recurrence from a 4.5 and 12 year old males respectively. Both tumors belonged to the SHH‐MB molecular group suggesting a possible predilection of hypermutator phenotype within SHH‐MB.^15^ Though, the exact biological and clinical implications of hypermutation in MB is yet to be systematically reviewed, a recent abstract published ISPNO indicated poor clinical outcomes in SHH‐MB with high mutational burden.^21^

A higher mutation rate in the coding region of a tumor genome is associated with generation of structurally and functionally altered epitopes or possible neoantigens.^22^ Neoantigens can trigger a rapid immunologic cytotoxic CD8+ T‐cell response often accompanied by several immune checkpoints to attenuate this effect.^16^ Therefore, hypermutation in tumors may indicate a sustained clinical response to immune checkpoint inhibition.

Furthermore, high mutation rates in tumors can lead to rapid generation of resistant clones when such tumors are treated with small molecule inhibitors (SMIs). This is very relevant to cases reported, as at least two of them belonged to SHH‐MB, the only subgroup for which there is a known SMI.^17^ More importantly, both POLE mutated tumors reported here were recurrent tumors. As there is no known therapy for patients with recurrent MBs, they are often considered for SMI therapy. The review of tumors with high TMB may respond better to immune checkpoint inhibitor therapy than SMIs.

The tumors with low mutation burden did not have mutational signature 10 contributions in their genome. Mutational signature analysis of the other tumors revealed high prevalence of HRD signatures 3 and 8. The initial study defining mutational signatures did not report the prevalence of HRD signatures in MB,^12^ however, subsequently two large genomic studies reported high prevalence of HRD signatures.^7^, ^16^ The clinical and biological relevance of high prevalence of the signatures 3 and 8 which are markers of homologous recombination defects^23^ requires further exploration and systematic study.

We conclude that hypermutations, though rare, are identified in MB and that mutational signature analysis may provide some useful insights into this disease. These observations are important and warrant further investigation since both could have therapeutic and prognostic implications in MB treatment.

## AUTHOR CONTRIBUTIONS

All authors had full access to the data in the study and take responsibility for the integrity of the data and the accuracy of the data analysis. Conceptualization, AB, SDJ, GWR; Methodology, AB, IB.; Investigation, AB,IB.; Formal Analysis, AB, IB; Resources, SDG, AC.; Writing ‐ Original Draft, AB, SDG; Writing ‐ Review & Editing, AB, GWR.; Visualization, AB, SDG, GWR; Supervision, SDG, AC, GWR.; Funding Acquisition; AB, SDG, AC.

## CONFLICT OF INTERESTS

The authors have stated explicitly that there are no conflicts of interest in connection with this article.

## ETHICAL STATEMENT

Use of participants' tissues in genetic studies, along with waiver of consent and waiver of HIPAA authorizations were approved by Institutional Review Boards of Van Andel Research Institute and Spectrum Health Helen DeVos Children's Hospital. Permission to download whole genome sequencing (WGS) data of the primary and recurrent MBs was obtained from the European Genome Archive (EGA).

## Supporting information


**Supplementary Figure 1** (A) Non‐parametric distribution and inter‐tumor variability of tumor mutational burden (TMB) among tumors. The Y‐axis depicts the mutations per Mb; the small black circles depict outlier cases with higher TMB. Outliers are calculated as any data points that lie beyond the point that is 1.5 times the interquartile range above the third quartile of distribution. Ends of the box represent the upper and lower quartiles; therefore, the box spans the interquartile range, and the median is marked by the dark horizontal line inside the box. Whiskers are the two lines outside the box that extend to the highest and the lowest observations. (B) Schematic plot demonstrating the functional derivatives of the POLE protein and location of mutations identified in T‐1. (C) Mutational signatures in non–*POLE* mutated tumors with higher mutational burden. Each individual bar represents each tumor. (D) Landscape of mutational signatures in non‐hypermutated medulloblastomas (MBs). The figure highlights the prevalence of signatures 3 and 8 in MBs, that is associated with homologous recombination defects in other solid tumors.

**Supplementary Figure 2** Copy number variation (CNV) landscape of T‐10. The diagram shows loss of chr7q and chr14q. Loss of Chr14q is a known high‐risk molecular feature in SHH‐MB.

Supplementary S1

## Data Availability

The whole genome sequencing data that support the findings of this study are available on request from the corresponding author upon a reasonable request. Whole exome sequencing data cannot be shared due to ethical reasons.
