# Supplementary material for: Rare cases of medulloblastoma with hypermutation
Source: Cancer Rep (Hoboken). 2021 Aug 5;5(5):e1521. doi: 10.1002/cnr2.1521 (PMC9124508; doi:10.1002/cnr2.1521)

Figure 2

A: Boxplot depicting non-paramentric distribution of TMB in analyzed tumors

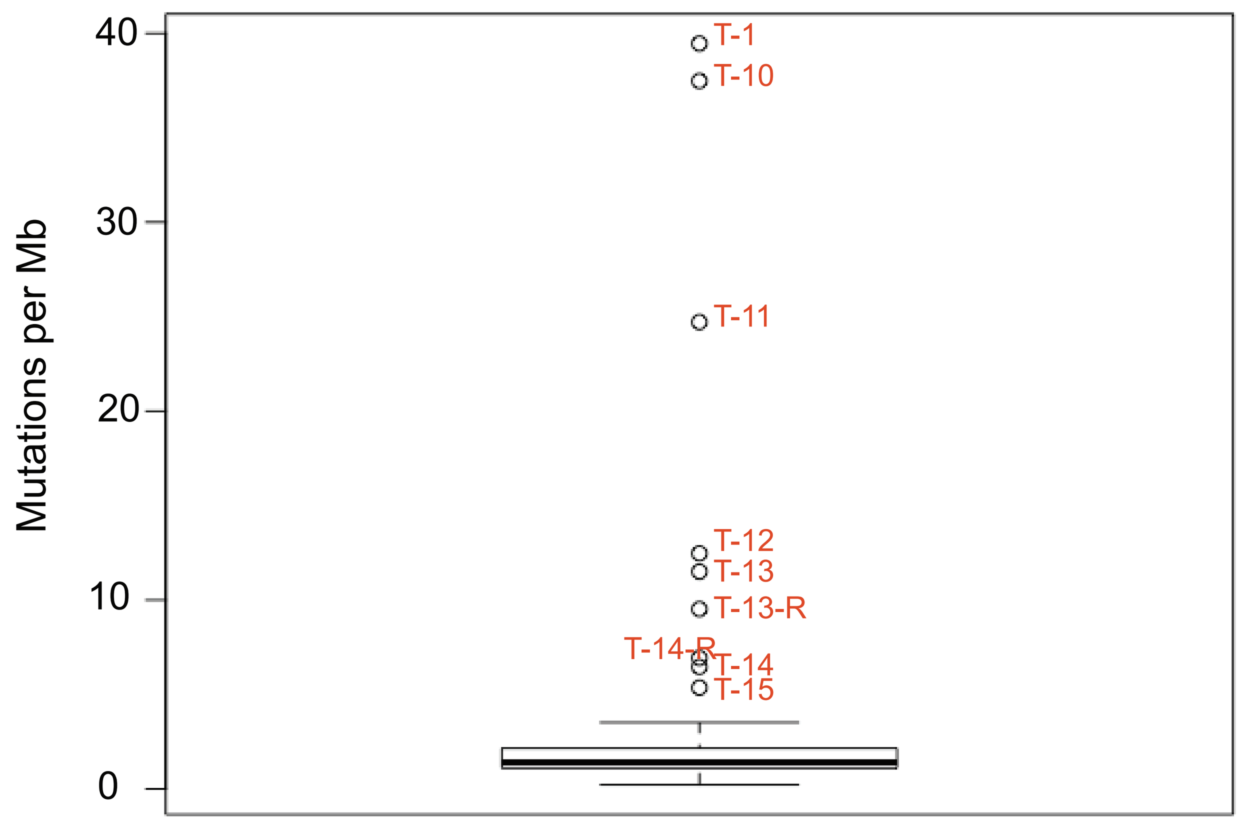

B: Functional derivative of POLE protein and locations of mutations identified in T-1

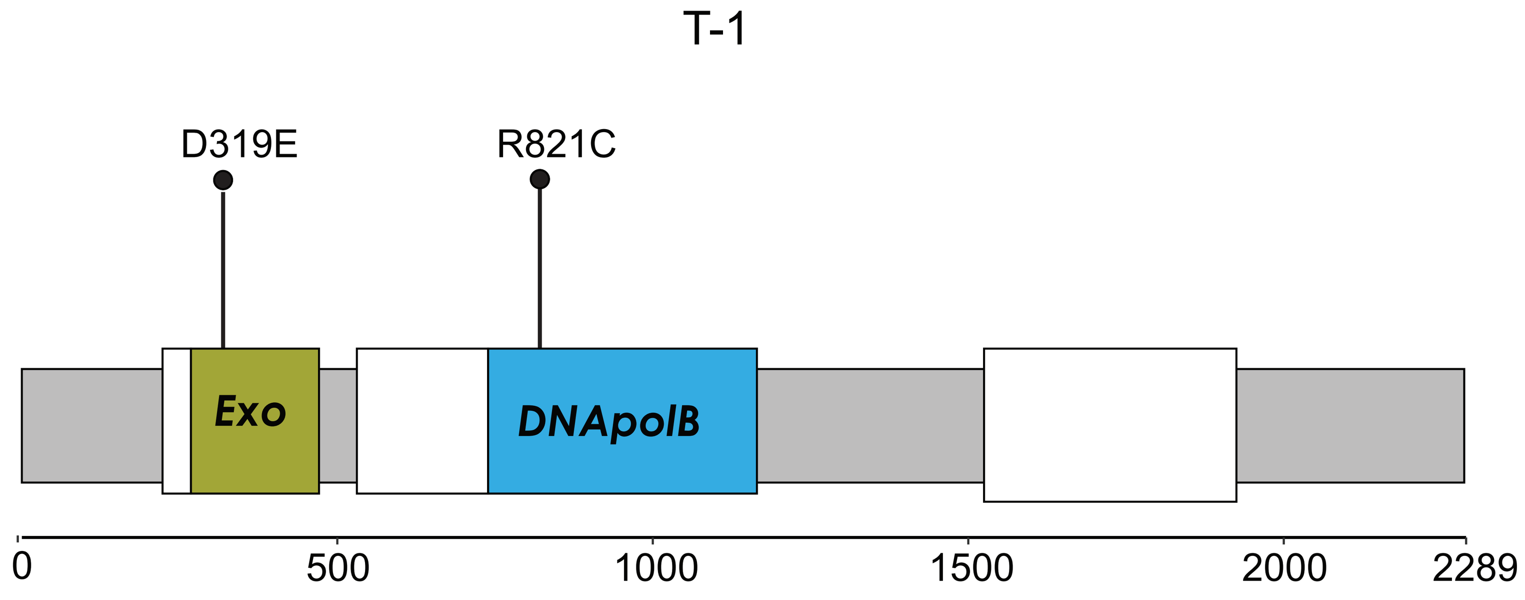

C: Mutational signatures in non POLE mutated hypermutated Medulloblastoma

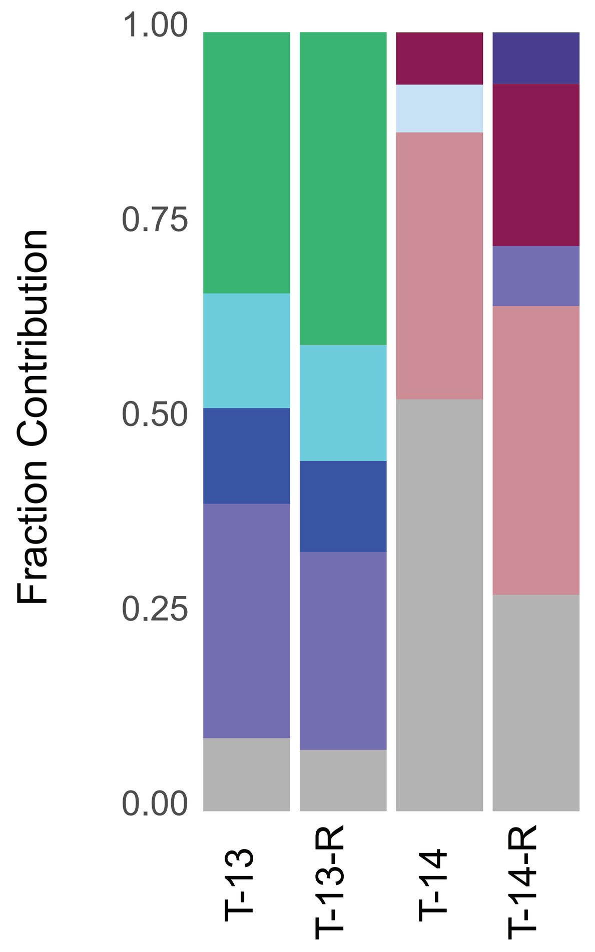

D: Mutational signatures in non-hypermutated Medulloblastoma

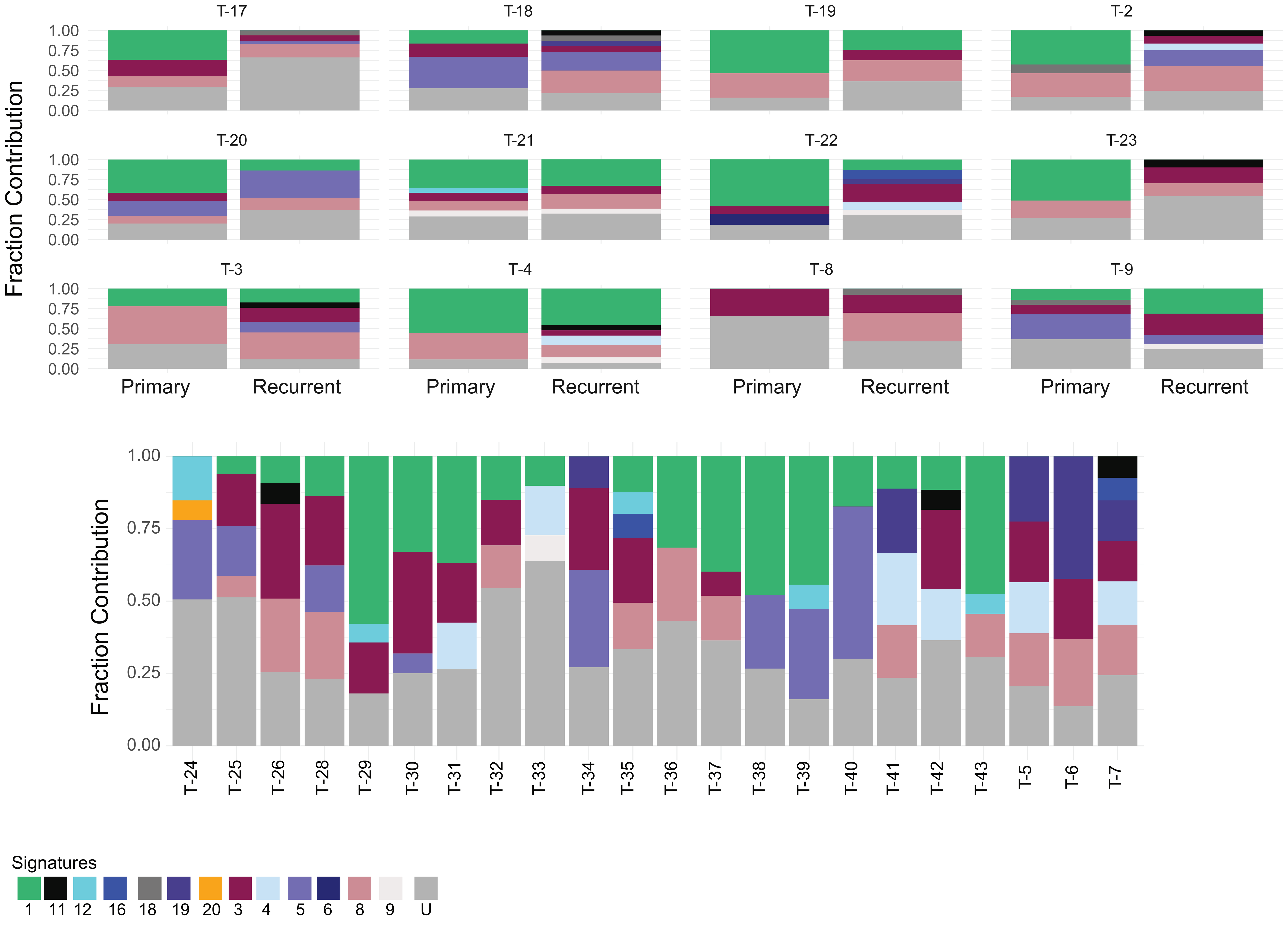

Supplement: Supplementary file 1 — Supplementary Figure 1 (A) Non‐parametric distribution and inter‐tumor variability of tumor mutational burden (TMB) among tumors. The Y‐axis depicts the mutations per Mb; the small black circles depict outlier cases with higher TMB. Outliers are calculated as any data points that lie beyond the point that is 1.5 times the interquartile range above the third quartile of distribution. Ends of the box represent the upper and lower quartiles; therefore, the box spans the interquartile range, and the median is marked by the dark horizontal line inside the box. Whiskers are the two lines outside the box that extend to the highest and the lowest observations. (B) Schematic plot demonstrating the functional derivatives of the POLE protein and location of mutations identified in T‐1. (C) Mutational signatures in non–POLE mutated tumors with higher mutational burden. Each individual bar represents each tumor. (D) Landscape of mutational signatures in non‐hypermutated medulloblastomas (MBs). The figure highlights the prevalence of signatures 3 and 8 in MBs, that is associated with homologous recombination defects in other solid tumors. [file CNR2-5-e1521-s003.pdf]
