# Supplementary figures and images for: Rare cases of medulloblastoma with hypermutation
Source: Cancer Rep (Hoboken). 2021 Aug 5;5(5):e1521. doi: 10.1002/cnr2.1521 (PMC9124508; doi:10.1002/cnr2.1521)

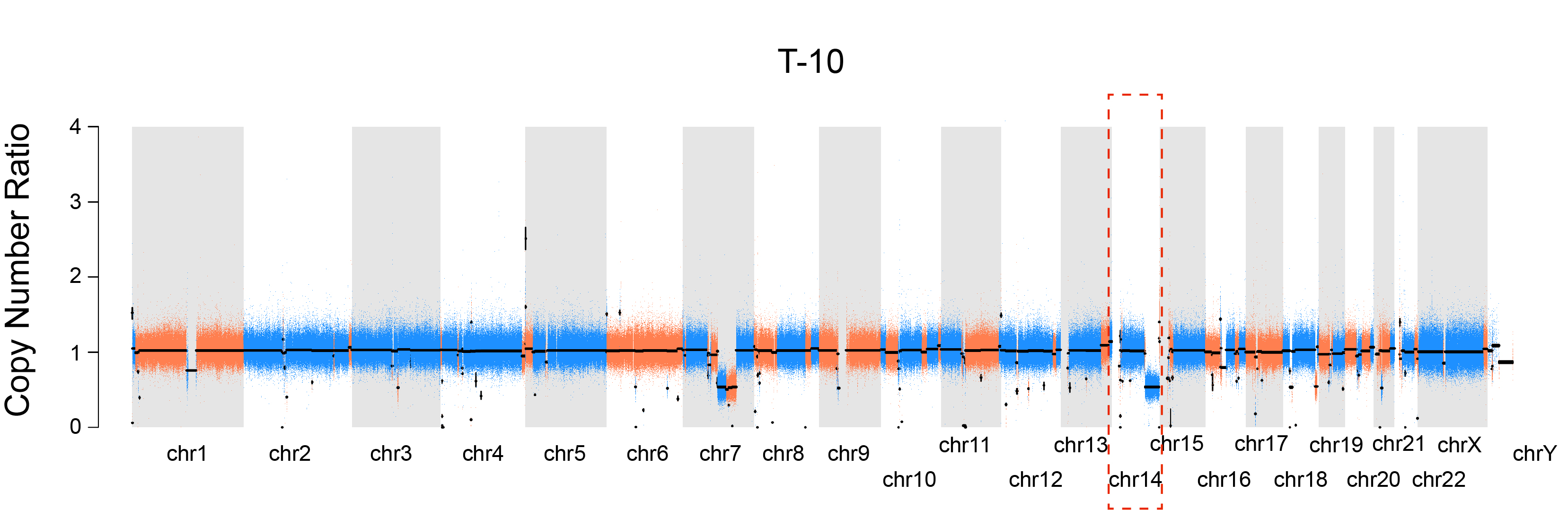

Supplement: Supplementary file 2 — Supplementary Figure 2 Copy number variation (CNV) landscape of T‐10. The diagram shows loss of chr7q and chr14q. Loss of Chr14q is a known high‐risk molecular feature in SHH‐MB. [file CNR2-5-e1521-s001.jpg]
