# Supplementary material for: Rare cases of medulloblastoma with hypermutation
Source: Cancer Rep (Hoboken). 2021 Aug 5;5(5):e1521. doi: 10.1002/cnr2.1521 (PMC9124508; doi:10.1002/cnr2.1521)
Supplement: Supplementary file 3 — Supplementary S1 [file CNR2-5-e1521-s002.docx]

**Supplementary Methods**

**Nucleic Acid Extraction:**

DNA was extracted from 50-100 microns of FFPE curls using the spin column-based nucleic acid extraction protocol as published and manufactured by Qiagen for nucleic acid extraction from FFPE tissue (Cat No. #56404). A spectrophotometric method (NanoDrop) was used to measure protein and organic contaminants in the DNA. The 260/280 ratio was used to determine protein contamination. As aromatic proteins have a strong UV absorbance at 280 nm, a high A 260/280 ratio between 1.8 and 2.1 was used as an acceptable purity. A 260/230 ratio was measured as an indicator of organic contaminants such as phenol and TRIzol.

The Advanced Analytical Fragment Analyzer was used to assess the degree of fragmentation in the gDNA material. The analysis revealed variable fragmentation within the samples. This result was expected, as the nucleic acid was extracted from archived FFPE samples. We did not reject any samples based on fragment lengths. We modified the library preparation protocol (see below) based on degree of fragmentation.

**Exome Capture and Sequencing:**

SureSelect^XT^ Clinical Research Exome V2 (Agilent Technologies) was used to capture exomes of tumor samples according to the manufacturer’s protocol, with modifications based on degree of DNA fragmentation. Briefly, DNA was sheared and samples with average fragment sizes less than 500 base pairs were not further sonicated. Sheared DNA ends were blunted and adenylated at the 3′ end. Adapters were ligated to ends of the DNA fragments to generate libraries complementary to sequencing primers on the flow cell. Additional sequences were added by tailed primers during PCR to selectively enrich DNA fragments that have adapter molecules on both ends and to amplify the amount of DNA in the library. The pre-capture adapter ligated libraries were hybridized with biotinylated SureSelect^XT^ RNA baits. These RNA baits specifically target the genomic regions linked to human diseases (genes, splice sites, non-coding exons, intronic variants, promoter regions, non-coding RNAs, and breakpoint spanning regions). The total sequencing region covered by the target capture baits was 67.2 Mb, which included more than 99% of genes included in The Consensus Coding Sequence (CCDS). Post-capture PCR was performed for 11 cycles. These libraries were then sequenced to an approximate depth of 300× coverage to compensate for any uneven genomic representation in the NovaSeq 6000 System - Illumina.

**Alignment, Variant Calling, Filtering, Annotation, and Analysis:**

As a quality control step, sequencing adapters and all bases with a Phred quality score of less than 30 were trimmed from the ends of reads using Trim Galore. Then, reads were aligned against the human reference genome (version GRCh38) with the Burrows–Wheeler Aligner^1^**.** Thirty-three exome capture samples in this cohort did not have matched germline controls. Somatic mutations were called using the “Tumor-Only” (TO) mode of Mutect2, part of the Genome Analysis Toolkit V4 (GATK)^2^. Variant annotation was performed after variant calling by using the Ensemble database with the help of the Variant Effect Predictor (VEP-Release-92) tool^3^. The VEP annotation tool uses CCDS for identifying genes in the variant call set. Following annotation and filtration, cataloged somatic mutations were analyzed in the R-computing environment (Supplementary Table 1). Signatures of somatic mutations were calculated by considering six major mutation classes (C>A, C> G, C>T, T>A, T>G, T>C) and then further dividing each of the six mutation types into 16 subtypes based on the 3′ and 5′ nucleotide. The contribution of known mutational signatures was calculated using deconstructSigs.

In the cohort of tested tumors, 53 cases were obtained from EGA. These data were in the form of BAM files of WGS aligned to GRCh37. To create consistency among datasets obtained from various sources, BAM files were downloaded from EGA, and reads were realigned to GRCh38 with somatic variants called using Mutect2 best practices guidelines published by the Broad Institute’s GATK (version 3.7).

**Copy Number Analysis:**

Copy number analysis was done using GATK v3.7 using their best practice workflow. Briefly, read coverage counts across genomic intervals was collected, standardized, and denoised. Segments were then modelled using the ModelSegments and finally CNAs were called using CallCopyRatioSegments. A panel of normals generated from the available germline samples was used in all cases. Segments were imported into R v4.0.2, and, based on the distribution of the segment copy number, filtered for an inferred copy number less than 0.5 for deletions and greater than 1.5 for amplifications. The filtered alterations were then intersected with genomic annotation for GRCh38 build using the packages GenomicRanges v1.42.0^4^ and BioMart v4.1, ^5^

We tested the difference in copy number "landscape" by testing for differences in the proportion of the genome amplified or deleted in the tumor genomes. Specifically, the proportion of each genome amplified and deleted was used in a beta regression to test for differences between hypermutated and non-hyper-mutated tumors. The result showed that the two hypermutated. tumors, T-1, and T-10, were not different from other samples for the proportion of their genome involved in copy number alterations.

**Statistical Analysis:**

Outliers for analysis were defined as any data point that lies beyond the point that is 1.5 times the interquartile range above the third quartile of the distribution (Supplementary Figure 1A).

**Supplementary Table 1: Important Computational Tools (R Packages)**

| ggplot2 | Elegant Graphics for Data Analysis; Hadley Wickham et al. |
| --- | --- |
| maftools | Maftools: efficient and comprehensive analysis of somatic variants in cancer^6^ |
| deconstructSigs | deconstructSigs: delineating mutational processes in single tumors to distinguish DNA repair deficiencies and patterns of carcinoma evolution^7^ |

**References:**

1. Li H. Aligning sequence reads, clone sequences and assembly contigs with BWA-MEM. *arXiv:13033997 [q-bio]*. Published online March 16, 2013. Accessed April 9, 2019. http://arxiv.org/abs/1303.3997

2. McKenna A, Hanna M, Banks E, et al. The Genome Analysis Toolkit: a MapReduce framework for analyzing next-generation DNA sequencing data. *Genome Res*. 2010;20(9):1297-1303. doi:10.1101/gr.107524.110

3. McLaren W, Gil L, Hunt SE, et al. The Ensembl Variant Effect Predictor. *Genome Biology*. 2016;17(1):122. doi:10.1186/s13059-016-0974-4

4. Lawrence M, Huber W, Pagès H, et al. Software for Computing and Annotating Genomic Ranges. *PLOS Computational Biology*. 2013;9(8):e1003118. doi:10.1371/journal.pcbi.1003118

5. Durinck S, Spellman PT, Birney E, Huber W. Mapping Identifiers for the Integration of Genomic Datasets with the R/Bioconductor package biomaRt. *Nat Protoc*. 2009;4(8):1184-1191. doi:10.1038/nprot.2009.97

6. Mayakonda A, Lin D-C, Assenov Y, Plass C, Koeffler HP. Maftools: efficient and comprehensive analysis of somatic variants in cancer. *Genome Res*. 2018;28(11):1747-1756. doi:10.1101/gr.239244.118

7. Rosenthal R, McGranahan N, Herrero J, Taylor BS, Swanton C. deconstructSigs: delineating mutational processes in single tumors distinguishes DNA repair deficiencies and patterns of carcinoma evolution. *Genome Biology*. 2016;17(1):31. doi:10.1186/s13059-016-0893-4
